# Global m6A RNA Methylation in SARS-CoV-2 Positive Nasopharyngeal Samples in a Mexican Population: A First Approximation Study

**DOI:** 10.3390/epigenomes6030016

**Published:** 2022-06-29

**Authors:** Jorge Luis Batista-Roche, Bruno Gómez-Gil, Gertrud Lund, César Alejandro Berlanga-Robles, Alejandra García-Gasca

**Affiliations:** 1 Molecular and Cellular Biology, Centro de Investigación en Alimentación y Desarrollo, Mazatlán 82112, Mexico; jbatista220@estudiantes.ciad.mx; 2 Microbial Genomics, Centro de Investigación en Alimentación y Desarrollo, Mazatlán 82112, Mexico; bruno@ciad.mx; 3 Department of Genetic Engineering, CINVESTAV Irapuato Unit, Irapuato 36821, Mexico; gertrud.lund@cinvestav.mx; 4 Environmental Management, Centro de Investigación en Alimentación y Desarrollo, Mazatlán 82112, Mexico; cesar@ciad.mx

**Keywords:** SARS-CoV-2, m6A methylation, nasopharyngeal samples, viral variants

## Abstract

The Severe Acute Respiratory Syndrome-Coronavirus-2 (SARS-CoV-2) is the causal agent of COVID-19 (Coronavirus Disease-19). Both mutation and/or recombination events in the SARS-CoV-2 genome have resulted in variants that differ in transmissibility and severity. Furthermore, RNA methylation of the N6 position of adenosine (m6A) is known to be altered in cells infected with SARS-CoV-2. However, it is not known whether this epitranscriptomic modification differs across individuals dependent on the presence of infection with distinct SARS-CoV-2 variants, the viral load, or the vaccination status. To address this issue, we selected RNAs (*n* = 60) from SARS-CoV-2 sequenced nasopharyngeal samples (*n* = 404) of 30- to 60-year-old outpatients or hospitalized individuals from the city of Mazatlán (Mexico) between February 2021 and March 2022. Control samples were non-infected individuals (*n* = 10). SARS-CoV-2 was determined with real-time PCR, viral variants were determined with sequencing, and global m6A levels were determined by using a competitive immunoassay method. We identified variants of concern (VOC; alpha, gamma, delta, omicron), the variant of interest (VOI; epsilon), and the lineage B.1.1.519. Global m6A methylation differed significantly across viral variants (*p* = 3.2 × 10^−7^). In particular, we found that m6A levels were significantly lower in the VOC delta- and omicron-positive individuals compared to non-infected individuals (*p* = 2.541236 × 10^−2^ and 1.134411 × 10^−4^, respectively). However, we uncovered no significant correlation between global m6A levels and viral nucleocapsid (*N*) gene expression or age. Furthermore, individuals with complete vaccination schemes showed significantly lower m6A levels than unvaccinated individuals (*p* = 2.6 × 10^−4^), and differences in methylation levels across variants in unvaccinated individuals were significant (*p* = 3.068 × 10^−3^). These preliminary results suggest that SARS-CoV-2 variants show differences in global m6A levels.

## 1. Introduction

SARS-CoV-2 is an enveloped, spheroidal-shaped virus that causes the disease known as COVID-19 in humans. It has a capsid that contains the structural proteins spike (S), membrane (M), envelope (E), and the nucleocapsid protein (N). Its genome is a single, positive stranded ribonucleic acid (RNA) that behaves as messenger RNA (mRNA) [1]. The N protein is a crucial structural component of SARS-CoV-2 that participates in the virion assembly and is often used as a diagnostic marker of viral infection [2]. In a vaccine setting, the N protein induces SARS-CoV-2-specific T cell proliferation and cytotoxic activity and promotes long-lasting T cell immunity [3].

The main receptor and route of entry of SARS-CoV-2 into the host cells is the angiotensin-converting enzyme 2 (ACE2), which binds with the S protein of SARS-CoV-2 and mediates the fusion of viral and cell membranes. ACE2 is found in the membranes of different human cells such as pneumocytes, enterocytes, proximal tubular cells of the kidney, and endothelial cells of veins and arteries [4]. Various mutations in the viral genome have given rise to SARS-CoV-2 variants, which differ not only in nucleotide sequences but also in transmissibility and severity [5,6].

Following the discovery of RNA methylation at N6- position of adenosine (m6A), the most prevalent modification of RNA in mammalian cells [7,8], this modification has also been detected in several viral genomes [9]. Most m6A is found in the consensus sequence motif DRACH (where A* denotes the methylated adenosine, D denotes A, G or U, R denotes A and G, and H denotes A, C or U) and mRNAs may contain from one to up to 20 m6A sites or more [10,11]. Genomic and viral levels of m6A are dynamically regulated by the activity of methyltransferase enzymes (i.e., “writers” such as METTL3, METTL14 that transfer methyl groups to nitrogen 6 of the adenosine) and demethylases (“erasers” such as fat mass and obesity-associated protein (FTO) and a ketoglutarate dependent dioxygenase homolog 5 (ALKBH5)). Once methylated, m6A is recognized by “reader” proteins (e.g., YTH N6-Methyladenosine RNA Binding Protein domain-containing proteins YTHDC1 and C2, YTHDF1, F2 and F3) that influence the fate of m6A RNA, either by guiding mRNAs to ribosomes for translation or to other complexes for their degradation [11,12].

It was recently reported that SARS-CoV-2 RNA, as well as negative-stranded viral RNAs, are dynamically methylated in vitro, as is the host cell RNA [13]. In human hepatocarcinoma cells (Huh7), SARS-CoV-2 genomic RNA is gradually methylated during infection and m6A occurs more frequently towards the 3′ region of the genome [14]. In addition, SARS-CoV-2 proteins interact with the host m6A methylation machinery to modulate viral replication. For example, an increase in METTL3 expression 48 h post-infection in Vero E6 cells was positively associated with SARS-CoV-2 replication [15]. In accordance, depletion of METTL3 leads to a reduction in SARS-CoV-2 replication [13,15,16], although the opposite has also been reported [14]. Furthermore, knockdown of specific erasers (ALKBH5) and readers (YTHDF1, YTHDF2, YTHDF3) have also been shown to impact on viral replication [14,16].

Recent studies show that SARS-CoV-2 infection changes the host cell m6A methylome in vitro, promoting differential expression of host genes [13], and in vivo, altering m6A modification levels in lymphocytes from peripheral blood samples by increased expression of the m6A methyltransferase RNA-binding motif protein 15 (*RBM15*) [17] or decreased expression of *METTL3* in epithelial cells of bronchoalveolar lavage fluid of COVID-19 patients [13].

To date, only a few studies have probed for alterations in global m6A levels in a limited number of COVID-19 positive individuals (*n* = 2–20) [13,17,18]. These studies found increased levels of m6A in peripheral blood samples [17] or in epithelial cells of bronchoalveolar lavage fluid [13] in infected individuals relative to non-infected individuals that are associated with increased expression of *RBM15*, which encodes for a protein that facilitates m6A by guiding METTL3 to the target RNA sequence [17]. To our knowledge, no studies have compared m6A levels across individuals infected with distinct COVID-19 variants. Therefore, we measured global levels of this modification in nasopharyngeal samples from adult (30- to 60-year-old) male and female patients infected with different SARS-CoV-2 variants between February 2021 and March 2022.

## 2. Results

### 2.1. Viral Variants Detected in Mazatlán, Mexico during the COVID-19 Pandemic (February 2021–March 2022)

**Figure 1:**
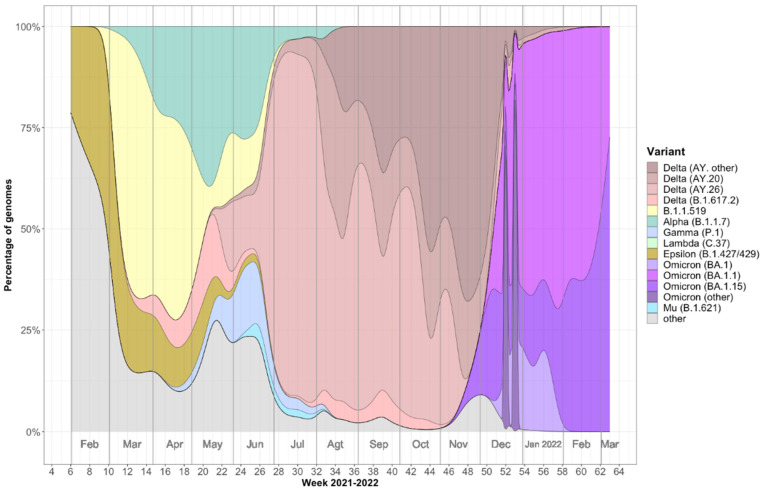
Percentage of genomes of SARS-CoV-2 variants sequenced in Mazatlán, Mexico, from February 2021 to March 2022 (*n* = 404). Delta (AY. other) = AY.100, AY.103, AY.113, AY.119, AY.122.4, AY.25, AY.3, AY.39, AY.43, AY.44. AY.53. Omicron (other) = BA.1.1.16, BA.1.1.2, BA.1.1.8, BA.1.13, BA.1.14, BA.1.17.

Analysis of nasopharyngeal samples from a total of 404 individuals (219 males and 185 females) revealed the presence of distinct SARS-CoV-2 variants during the second, third, and fourth waves of the COVID-19 pandemic in Mazatlán, Mexico from February 2021 to March 2022 (Figure 1). During the second wave, epsilon and B.1.1.519 lineage variants predominated in the population, peaking between February and March 2021. During the third wave, peaking between June and August, the delta variant dominated, although the alpha and gamma variants were also detected. Finally, the fourth wave, peaking between January and February, was dominated by the omicron variant.

### 2.2. Global m6A Levels and Nucleocapsid (N) Gene Expression in Nasopharyngeal Samples

**Figure 2:**
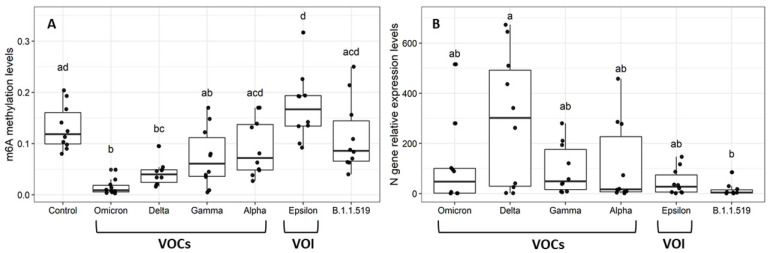
(**A**) Global m6A levels of RNAs extracted from human nasopharyngeal samples, tested for SARS-CoV-2 by real-time PCR based on viral variant (*n* = 10 each). Control: SARS-CoV-2 negative samples (Kruskal-Wallis *p* = 3.2 × 10^−7^); different letters indicate significant differences in methylation levels. (**B**) Relative expression of the *N* gene in RNAs extracted from human nasopharyngeal samples, tested for SARS-CoV-2 by real-time PCR based on viral variant (*n* = 10 each) (Kruskal-Wallis, *p* = 0.041; Dunn, *p* = 0.024); different letters indicate significant differences in gene expression levels. VOC: omicron, delta, gamma, and alpha. VOI: epsilon; B.1.1.519: lineage found in Mexico during the second wave.

Global m6A levels were determined with 60 randomly selected sequenced samples of each of the following variants: omicron (BA.1), delta (B.1.617.2), gamma (P.1), alpha (B.1.1.7), and epsilon (B.1.429), as well as the B.1.1.519 lineage (Figure 2A). Table 1 shows the average age, sex distribution, and vaccination status of individuals in each variant group. Vaccination data were available for 64 of the 70 samples analyzed; 8 and 4 samples were from patients with a complete or partial vaccination schemes, respectively, while 52 samples came from unvaccinated patients.

A Kruskal–Wallis test revealed significant differences in global m6A according to viral variant (*p* = 3.2 × 10^−7^). In particular, delta and omicron-positive patients showed significantly lower m6A levels than the control group (SARS-CoV-2 negative) (*p* = 2.541236 × 10^−2^ and 1.134411 × 10^−4^ for delta and omicron respectively).

**Figure 3:**
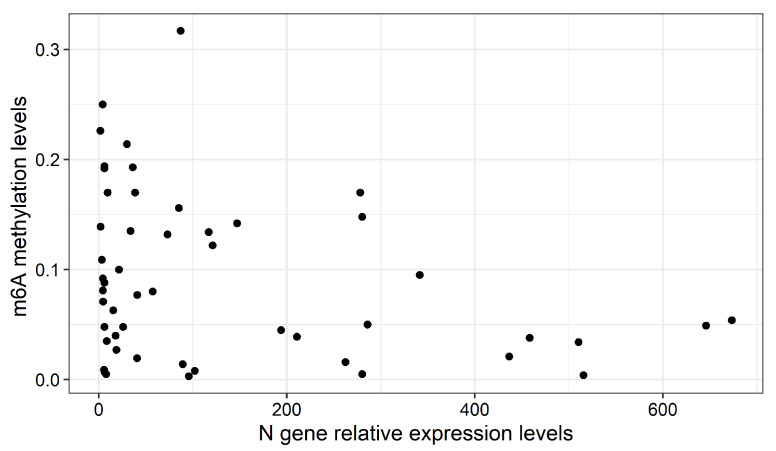
Scatter plot between relative expression of the *N* gene and m6A levels (Spearman’s rank correlation, rho = −0.063, *p* = 0.63).

To understand whether the differences in variant m6A levels were related to viral load, we compared expression levels of the viral nucleocapsid (*N*) gene across variants. Only delta and B.1.1.519 variants showed significant differences in *N* expression levels (Kruskal-Wallis, *p* = 4.1 × 10^−2^; Dunn, *p* = 2.4 × 10^−2^) (Figure 2B). However, we found no significant correlation, neither between m6A levels and relative expression of the *N* gene of all, or only unvaccinated individuals (r = −0.06, *p* = 0.63 and r = 0.004, *p* = 0.9803, respectively) (Figure 3), nor between m6A and age (r = 0.15, *p* = 0.21).

**Figure 4:**
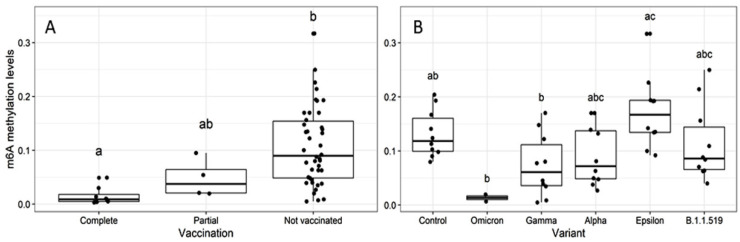
(**A**) Global m6A levels in of RNAs extracted from human nasopharyngeal samples, tested for SARS-CoV-2 by real-time PCR with different vaccination schemes (*n* = 64). Partial and complete vaccination refers to the application of one or two doses, respectively, of authorized vaccines in Mexico (AZD1222, CoronaVac, BNT162b2, Ad5-nCOV, and mRNA-1273 from the companies AstraZeneca, Sinovac, Pfizer-BioNTech, CanSinoBio, and Moderna, respectively) (Kruskal-Wallis, *p* = 2.6 × 10^−4^; Dunn test, *p* = 2.5 × 10^−4^); *n* = 8, 4, and 52 for complete, partial, and unvaccinated individuals, respectively. (**B**) Global m6A levels of RNAs extracted from human nasopharyngeal samples from unvaccinated patients, tested for SARS-CoV-2 by real-time PCR based on viral variant (*n* = 52). Control: SARS-CoV-2 negative samples (Kruskal–Wallis *p* = 3.068 × 10^−3^); different letters indicate significant differences in methylation levels.

Finally, we evaluated whether patient vaccination status affected global m6A RNA methylation levels. Samples from patients with complete vaccination schemes showed significantly lower levels of RNA methylation compared to unvaccinated patients (Kruskal-Wallis, *p* = 2.6 × 10^−4^; Dunn test, *p* = 2.5 × 10^−4^) (Figure 4A). Importantly, samples from vaccinated patients were obtained during the third and fourth waves, dominated by delta and omicron variants, respectively. To exclude that vaccination status affected the observed differences between variants, we performed a Kruskal-Wallis analysis restricted to unvaccinated individuals. As previously observed, m6A levels differed significantly across variants (*p* = 3.068 × 10^−3^), with omicron and some gamma-infected patients showing the lowest methylation levels (Figure 4B). The delta variant was not represented in unvaccinated individuals.

## 3. Discussion

RNA modifications such as m6A play an important role in different physiological processes and human diseases. In the innate immune response, m6A-modfied mRNAs are essential for the translation of co-stimulatory molecules that allow dendritic cell maturation and subsequent T cell activation [19]. Also, the immunosuppressive functions of regulatory T cells are maintained by m6A-driven degradation of the suppressor of cytokine signaling (SOCS) transcripts [8]. In addition, RNAs from several viral families acquire m6A modifications as a common strategy to evade innate host immunity [20].

It has been reported that m6A deficiency in the vesicular stomatitis virus (VSV) genome triggers significantly higher levels of type I interferon [20]. However, some m6A sites of viral RNAs favor the evasion of the pattern recognition receptors of the cellular innate immune response and promote viral replication [21]. Conversely, certain transcripts of some RNA viruses and adenoviruses contain m6A-methylated sites that negatively influence infection [14]. Currently, there is a controversy in the literature regarding the effects of m6A methyltransferases on SARS-CoV-2 replication. Apparently, depending on the cell type, depletion of m6A methyltransferases can decrease viral load [13] or increase viral replication and the percentage of SARS-CoV-2 infected cells [14].

To our knowledge, this is the first report showing that global m6A levels of nasopharyngeal RNA samples of patients infected with SARS-CoV-2 differ among viral variants. Interestingly, the two most contagious variants (delta and omicron)—with omicron being the most contagious globally—[22] showed the lowest methylation levels. These differences are unlikely to reflect variation in disease severity across variants, as COVID-19 patients with beta and delta variants generally are at higher risk of developing severe disease compared to patients with alpha, gamma [23], and omicron variant(s), whose mutations have been suggested to contribute to the host immune escape [24]. Interestingly, DRACH motifs are highly conserved among SARS-CoV-2 variants [25], thus the contribution of DRACH motifs to differential methylation levels among variants remains unclear and require further research.

From a public health perspective, identifying a reliable marker of severe COVID-19 risk is of utmost importance. Unfortunately, we have no information regarding disease severity of the individuals analyzed in this study since most samples were from outpatients. In addition, we found no correlation between viral load and m6A levels across variants. However, previous studies have reported a higher m6A level in lung or peripheral blood from patients with moderate or severe COVID-19 disease compared to healthy individuals [13,17,18,26]. Interestingly, in peripheral blood these changes were associated with increased expression of *RBM15* [17]. Moreover, Qiu et al. [26] proposed a predictive “m6A score” to quantify and model the m6A pattern in blood leukocytes for each COVID-19 patient based on m6A levels and nine selected differentially expressed genes (m6A-DEGs) mostly related to the immune response; in this model, patients displaying higher (protective) scores showed a better prognosis related to T-cell activation compared to patients with lower scores; in addition to clinical prognosis, the model may predict the possibility of contracting COVID-19 in patients infected with SARS-CoV-2, as well as the detection of SARS-CoV-2 carriers [26].

Our study also revealed significantly lower levels of m6A in samples from fully vaccinated compared to unvaccinated individuals (*n* = 8 and 52, respectively). Most unvaccinated individuals were positive for alpha, gamma, epsilon, and B.1.1.519 variants, whereas delta and omicron were the dominant variants in vaccinated individuals. Importantly, we show that the variation in m6A levels across variants, in particular the low levels in delta and omicron, could not be explained by vaccination status alone. While changes in DNA methylation levels have been reported after influenza vaccination [27,28], there is no information regarding RNA methylation after vaccination. Potential vaccination-mediated m6A is intriguing and deserves further investigation.

The delta variant showed significant differences in the mean levels of *N* expression when compared with the B.1.1.519 variant, indicating that the delta variant had the highest viral loads. Similarly, additional studies show that patients infected with the delta variant presented increased viral loads compared to other SARS-CoV-2 variants [29,30]. Conversely, a recent study that compared a larger number of individuals positive for alpha or delta to other variants (*n* = 36 and 41, respectively), neither found significant differences between mean viral load across variants, nor between vaccinated and unvaccinated participants (37 vaccinated and 136 unvaccinated) [31]. In our study, no significant differences in *N* gene expression were observed among VOC- and VOI-positive samples, consistent with the previous report.

The results presented in this study are preliminary and should be interpreted with caution due to the following limitations. First, although all patients enrolled in this study were Mexican and living in the city of Mazatlán, genetic background, health and immune conditions, as well as lifestyle, greatly differ across individuals. Furthermore, we received samples from different hospitals which may differ in the sampling process and/or the social/economic status of the patients. Second, global m6A data represented the sum of methylation of both human and viral RNAs; therefore, additional studies are needed to determine their relative contributions. Third, due to the small amounts of RNA obtained from each nasopharyngeal sample, we were unable to perform technical duplicates of m6A content. Fourth, since we only had access to amplification data of the viral *N* and human *Rp* genes, calculations of viral *N* expression were performed relative to *Rp*. Fifth, increasing the sample size of analyzed individuals is important in order to improve the reliability of the conclusions presented.

**Figure 5:**
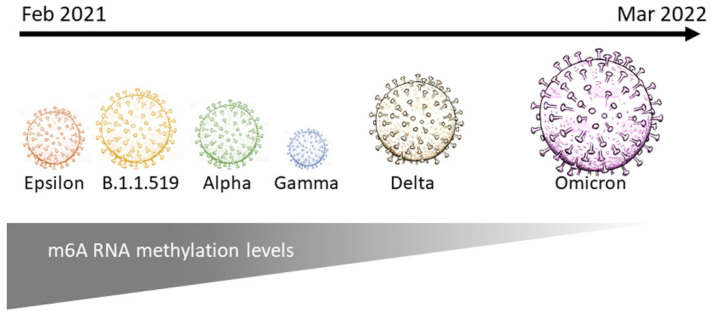
Schematic representation of m6A levels (grey triangle) among different SARS-CoV-2 variants from February 2021 to March 2022. Circle size represents number of cases per variant (smaller circles = less cases; larger circles = more cases) in the studied population.

The port of Mazatlán is a very touristic location, receiving thousands of visitors from Mexico and other countries around the world every year. Rapid changes of the dominant variants over the sampling period could be due to the crowded environment allowing the virus to spread. In addition, high mutation and recombination rates of RNA viruses, as well as asymptomatic infections, could also contribute to the rapid diversification of SARS-CoV-2 [32] even among fully vaccinated individuals. Despite the limitations of this study (which can be addressed in future studies), our results suggest that viral variants modulate genome and/or viral m6A levels differentially (Figure 5). Further research is needed to fully unravel and understand the molecular basis of these differences and its relevance in viral infection and transmission, as well as the implications in human immunity and health.

## 4. Materials and Methods

### 4.1. Samples

This study used 404 SARS-CoV-2 PCR-positive sequenced samples from the National Epidemiological Surveillance Consortium (see Figure 1), from which 60 samples (plus 10 samples from SARS-CoV-2 negative patients) were randomly selected for further analysis. Patients <30 or >60 years old, as well as SARS-CoV-2 PCR-positive samples with Cts <30 were excluded.

### 4.2. Identification and Sequencing of SARS-CoV-2 Positive Samples

Nasopharyngeal samples were taken by health professionals from different hospitals in Mazatlán (Mexico) and transported to the Molecular Diagnostic Laboratory at the Research Centre for Nutrition and Development (LDM-CIAD, official authorization number: DGE-DDYR-DSAT-04471-2020) for SARS-CoV-2 detection by real-time PCR (2019-nCov CDC^®^ Integrated DNA Technologies, Coralville, IA, USA). Relative expression levels of the viral *N* gene were determined for each sample as follows [33]:

(1) $$\Delta C_{T}=\frac{C_{T}N1+C_{T}N2}{2}-C_{T}Rp$$

where *N*1 and *N*2 are regions of the viral *N* gene and *Rp* is the human *RNAse P* gene (Supplementary File S1).

RNA samples (*n* = 404) with higher *N* levels (*C_T_* < 30) were selected for sequencing through the National Epidemiological Surveillance Consortium (COVIGEN). Complementary DNAs (cDNAs) were synthesized from total RNAs using the GoScript™ Reverse Transcription System (Promega, Madison, WI, USA). Libraries were prepared for sequencing using the COVID-Seq test (Illumina, San Diego, CA, USA), and the MiniSeq^TM^ sequencing system.

### 4.3. Global RNA Methylation Assay

Sequenced SARS-CoV-2-positive RNA samples (*n* = 60; 10 per variant) were randomly selected from adult (30–60 years old) patients (men and women). In addition, 10 negative samples were used as controls. RNA concentration and purity were measured with a spectrophotometer (Nanodrop, Thermo Fisher Scientific, Waltham, MA, USA Thermo). Because of the small amounts of RNA in nasopharyngeal samples, all samples were vacuum-lyophilized (LABCONCO, Kansas City, MO, USA) at −56 °C and 0.123 mBar, for 48 h. The samples were then resuspended in nuclease-free water for a final concentration of 60 ng/µL. The methylation assay was performed with the EpiQuik^TM^ m6A RNA Methylation Quantification Kit (Epigentek, Farmingdale, NY, USA), according to the manufacturer’s instructions. Absorbance at 450 nm was measured in a microplate reader (EPOCH2, BioTek, Thermo Fisher Scientific, Waltham, MA, USA). The standard curve was obtained and the relative methylation level *m*6*A* (%) was determined according to the following formula:

(2) $$m6A\;\left(\%\right)=\frac{\left(OD_{sample}-OD_{NC}\right)÷S}{\left(OD_{PC}-OD_{NC}\right)÷P}\times 100$$

where *OD_sample_* is the optical density of the sample, *OD_NC_* is the optical density of the negative control, *OD_PC_* is the optical density of the positive control, *S* is the amount of RNA (ng) applied to the well, and *P* is the amount of RNA (ng) applied to the positive control well (Supplementary File S2).

### 4.4. Statistical Analysis

Normality and homoscedasticity were evaluated with Shapiro-Wilk and Leneve tests, respectively. Kruskal–Wallis and *post-hoc* Dunn tests were performed with relative m6A RNA methylation and *N* gene expression data to detect differences among variants. Spearman correlations between relative m6A methylation and relative expression levels of the *N* gene or age were also done. In addition, differences in m6A methylation levels among vaccination schemes were analyzed by Kruskal–Wallis (unbalanced) and Dunn tests. The significance level was 0.05. All analyses were performed in RStudio 4.0.

## Figures and Tables

**Table 1:** Characteristics of patients (average age, sex distribution, and vaccination status) included in each variant group. The Control group refers to SARS-CoV-2 negative patients.

Variant	Average Age ± SD	Sex Distribution (*n* = 70)		Vaccination Status (*n* = 64)
		Males	Females	
Control	46 ± 7	5	5	Not vaccinated (*n* = 10)
Omicron	42 ± 8	5	5	Complete (*n* = 8) Not vaccinated (*n* = 2)
Delta	39 ± 10	5	5	Partial (*n* = 4)
Gamma	44 ± 7	5	5	Not vaccinated (*n* = 10)
Alpha	49 ± 8	5	5	Not vaccinated (*n* = 10)
Epsilon	45 ± 10	5	5	Not vaccinated (*n* = 10)
B.1.1.519	47 ± 11	5	5	Not vaccinated (*n* = 10)

## Data Availability

Data supporting reported results are provided as supplementary files.

## Supplementary Materials

The following supporting information can be downloaded at: https://www.mdpi.com/article/10.3390/epigenomes6030016/s1, Supplementary File S1: Real-time PCR data; Supplementary File S2: m6A RNA methylation data.
